# Supplementary material for: Comparative genomics provides new insights into the diversity, physiology, and sexuality of the only industrially exploited tremellomycete: Phaffia rhodozyma
Source: BMC Genomics. 2016 Nov 9;17:901. doi: 10.1186/s12864-016-3244-7 (PMC5103461; doi:10.1186/s12864-016-3244-7)
Supplement: Additional file 6: — List of orphan genes with links to PFAM (related to Additional file 1: Table S1). (ZIP 1428 kb) [file 12864_2016_3244_MOESM6_ESM.zip › BLAST_HTML_FTR/G00252_P.html]

BLAST Search Results


```
BLASTP 2.2.27+


Reference:
Stephen F. Altschul, Thomas L. Madden, Alejandro A. Schäffer,
Jinghui Zhang, Zheng Zhang, Webb Miller, and David J. Lipman (1997),
"Gapped BLAST and PSI-BLAST: a new generation of protein database
search programs", Nucleic Acids Res. 25:3389-3402.


Reference for
composition-based statistics:
Alejandro A. Schäffer, L. Aravind, Thomas L. Madden, Sergei
Shavirin, John L. Spouge, Yuri I. Wolf, Eugene V. Koonin, and
Stephen F. Altschul (2001), "Improving the accuracy of PSI-BLAST
protein database searches with composition-based statistics and
other refinements", Nucleic Acids Res. 29:2994-3005.


Database: nr
           71,551,133 sequences; 26,053,659,533 total letters


Query= G00252_P

Length=1137
                                                                      Score     E
Sequences producing significant alignments:                          (Bits)  Value

emb|CDZ98288.1|  hypothetical protein [Xanthophyllomyces dendrorh...  1849    0.0  


 >emb|CDZ98288.1| hypothetical protein [Xanthophyllomyces dendrorhous]
Length=1109

 Score = 1849 bits (4789),  Expect = 0.0, Method: Compositional matrix adjust.
 Identities = 1102/1136 (97%), Positives = 1105/1136 (97%), Gaps = 27/1136 (2%)

Query  1     MPDFPPTLPVLNSTSRPESPMGFVNGSHTHQPTSLQYTAPTQTSPRDHSSSSLVESDMLT  60
             MPDFPPTLPVLNSTSRPESPMGFVNGSHTHQPTSLQYTAPTQTSPRDHSSSSLVESDMLT
Sbjct  1     MPDFPPTLPVLNSTSRPESPMGFVNGSHTHQPTSLQYTAPTQTSPRDHSSSSLVESDMLT  60

Query  61    VKTPANTASGKPSSEIRPSSVYPPEVRSVVKHSSVNFVDHPVRPTPSPGIQSARIDSKQR  120
             VKTPANTASGKPSSEIRPSSVYPPEVRSVVKHSSVNFVDHPVRPTPSPGIQSARIDSKQR
Sbjct  61    VKTPANTASGKPSSEIRPSSVYPPEVRSVVKHSSVNFVDHPVRPTPSPGIQSARIDSKQR  120

Query  121   YESDLQILRRIKLDISSGHHTSYSAIIKPLALKSVSVTAEEAESQHSAATGLPPASSIHA  180
             YESDLQILRRIKLDISSGHHTSYSAIIKPLALKSVSVTAEEAESQHSAATGLPPASSIHA
Sbjct  121   YESDLQILRRIKLDISSGHHTSYSAIIKPLALKSVSVTAEEAESQHSAATGLPPASSIHA  180

Query  181   LPSTHRPKDSSLSTTTEKAVETVLLPDTDEPIEILTDSSTSSTPASIPSITAPRSVLASR  240
             LPSTHRPKDSSLSTTTE AVETVLLPDTDEPIEILTDSSTSSTPASIPSITAPRSVLASR
Sbjct  181   LPSTHRPKDSSLSTTTETAVETVLLPDTDEPIEILTDSSTSSTPASIPSITAPRSVLASR  240

Query  241   IIGNGPPVPTGPSALKDRLLSANHENASRGPSTNIAKFDPSVLPPTQPRAHAEGAAGRRQ  300
             IIGNGPPVPTGPSALKDRLLSANHENASRGPSTNIAKFDPSVLPPTQPRAHAEGAAGRRQ
Sbjct  241   IIGNGPPVPTGPSALKDRLLSANHENASRGPSTNIAKFDPSVLPPTQPRAHAEGAAGRRQ  300

Query  301   KRPKVDLDSSRDGPNAMMLDYSGMYDEVPAAPATAGTVDVSKVAGPDTTSEVMIKSSKKV  360
             KRPKVDLDSSRDGPNAMMLDYSGMYDEVPAAPATAGTVDVSKVA PDTTSEVMIKSSKKV
Sbjct  301   KRPKVDLDSSRDGPNAMMLDYSGMYDEVPAAPATAGTVDVSKVAVPDTTSEVMIKSSKKV  360

Query  361   APAAHQTSFHPTASTTTTTAAIKAAPLSSNDASSLHVQSQPAHTKPHELRSNISPAQPII  420
             APAAHQTSFHPTA+TTTTTAAIKAAPLSSNDASSLHV+SQPAHTKPHELRSNISPAQPII
Sbjct  361   APAAHQTSFHPTATTTTTTAAIKAAPLSSNDASSLHVRSQPAHTKPHELRSNISPAQPII  420

Query  421   SDSPTGGLPHGGADEEQGDQMDEEEAEIETFLTSCIEDSSATVDSGKSSFFVFQLFICRK  480
             SDSPTGGLPHGGADEEQGDQMDEEEAEIETFLTSCIEDSSATVDS               
Sbjct  421   SDSPTGGLPHGGADEEQGDQMDEEEAEIETFLTSCIEDSSATVDS---------------  465

Query  481   LIVRFDSLAYIDTERVDLSKSTPINTTSRSASSSSTSSSTSSINKSNSPSINPTEIEKDG  540
                        DTERVDLSKSTPINTTSRSASSSSTSSSTSSINKSNSPSINPTEIEKDG
Sbjct  466   -----------DTERVDLSKSTPINTTSRSASSSSTSSSTSSINKSNSPSINPTEIEKDG  514

Query  541   RIQMSIFEPRKLVSSIVQLSTNSVPTPIPVVSSAISSWWNWRVPLMRSRAQEPKTDSESV  600
             RIQMSIFEPRKLVSSIVQLSTNSVPTPIPVVSSAISSWWNWRVPLMRSRAQEPKTDSESV
Sbjct  515   RIQMSIFEPRKLVSSIVQLSTNSVPTPIPVVSSAISSWWNWRVPLMRSRAQEPKTDSESV  574

Query  601   VSPIITEVPPASSSSSSSSSLHSVTGEKDALKKVTKEASREQEEKEERERLALRTKRKKR  660
             VSPIITEVPPASSSSSSSS LHSVTGEKDALKKVTKEASREQEEKEERERLALRTKRKKR
Sbjct  575   VSPIITEVPPASSSSSSSS-LHSVTGEKDALKKVTKEASREQEEKEERERLALRTKRKKR  633

Query  661   ELEQQQYERVPDRPGLTNRRGPKQALKEKERRDREREAKDYKEARLNPMDKGKERSLGKG  720
             ELEQQQYERVPDRPGLTNRRGPKQALKEKERRDREREAKDYKEARLNPMDKGKERSLGKG
Sbjct  634   ELEQQQYERVPDRPGLTNRRGPKQALKEKERRDREREAKDYKEARLNPMDKGKERSLGKG  693

Query  721   GDRYHPYERSTNGLTSPNKSTSLHSRLASRGSELPARSFDLSSDRRPEDKFPYRYDVSDV  780
             GDRYHPYERSTNGLTSPNKSTSLHSRLASRGSELPARSFDLSSDRRPEDKFPYRYD SDV
Sbjct  694   GDRYHPYERSTNGLTSPNKSTSLHSRLASRGSELPARSFDLSSDRRPEDKFPYRYDASDV  753

Query  781   NADIISAKPNVHGAKTNARSVSLGLNDNYRPGKAQGIEIIRDDLSIRQYPKPFSPSPVHA  840
             NADIISAKPNVHGAKTNARSVSLGLNDNYRPGKAQGIEIIRDDLSIRQYPKPFSPSPVHA
Sbjct  754   NADIISAKPNVHGAKTNARSVSLGLNDNYRPGKAQGIEIIRDDLSIRQYPKPFSPSPVHA  813

Query  841   HAHVPPPAYQSGPVQSSAYPFRYQPPGPHVPPLPPPPIMDPPSTGYTRNKMSNGHNESSD  900
             HAHVPPPAYQSGPVQSSAYPFRYQPPGPH+PPLPPPPIMDPPSTGYTRNKMSNGHNESSD
Sbjct  814   HAHVPPPAYQSGPVQSSAYPFRYQPPGPHLPPLPPPPIMDPPSTGYTRNKMSNGHNESSD  873

Query  901   WSNTVRHAPAGYRDPFPHRQQLLPQQQQQHPPPPAHPIYPHADPSTLSRHPSSSFEPLPN  960
             WSNTVRHAPAGYRDPFPHRQQLLPQQQQQHPPPPAHPIYPHADPSTLSRHPSSSFEPLPN
Sbjct  874   WSNTVRHAPAGYRDPFPHRQQLLPQQQQQHPPPPAHPIYPHADPSTLSRHPSSSFEPLPN  933

Query  961   GNRTINNTSASRPGPNDSNLEILREMQALKEKLAMLEQQASGSGGRGGDASSAPLHTLPI  1020
             GNRTINNTSASRPGPNDSNLEILREMQALKEKLAMLEQQASGSGGRGGDASSAPLHTLPI
Sbjct  934   GNRTINNTSASRPGPNDSNLEILREMQALKEKLAMLEQQASGSGGRGGDASSAPLHTLPI  993

Query  1021  PPPHPQPRPYVHHEQQHQHIQQEQHRYQQGYPPYGAPPFAPSQSSRVRNLPPRTFEGGQE  1080
             PPPHPQP PYVHHEQQHQHIQQEQHRYQQGYPPYGAPPFAPSQSSRVRNLPPRTFEGGQE
Sbjct  994   PPPHPQPCPYVHHEQQHQHIQQEQHRYQQGYPPYGAPPFAPSQSSRVRNLPPRTFEGGQE  1053

Query  1081  KGWTATRLEGGLGGRFSDQSVPPAYQLQQPPPPPPPQQRDQTWAREDEESRRRGWR  1136
             KGWTATRLEGGLGGRFSDQSVPPAYQLQQPPPPPPPQQRDQTWAREDEESRRRGWR
Sbjct  1054  KGWTATRLEGGLGGRFSDQSVPPAYQLQQPPPPPPPQQRDQTWAREDEESRRRGWR  1109


Lambda      K        H        a         alpha
   0.309    0.126    0.366    0.792     4.96 

Gapped
Lambda      K        H        a         alpha    sigma
   0.267   0.0410    0.140     1.90     42.6     43.6 

Effective search space used: 14100816587325


  Database: nr
    Posted date:  Sep 23, 2015 12:05 AM
  Number of letters in database: 26,053,659,533
  Number of sequences in database:  71,551,133


Matrix: BLOSUM62
Gap Penalties: Existence: 11, Extension: 1
Neighboring words threshold: 11
Window for multiple hits: 40
```
